# A Novel Characterization of Amalgamated Networks in Natural Systems

**DOI:** 10.1038/srep10611

**Published:** 2015-06-02

**Authors:** Victor J. Barranca, Douglas Zhou, David Cai

**Affiliations:** 1 Courant Institute of Mathematical Sciences & Center for Neural Science, New York University; 2 NYUAD Institute, New York University Abu Dhabi; 3 Department of Mathematics, MOE-LSC, and Institute of Natural Sciences, Shanghai Jiao Tong University.

## Abstract

Densely-connected networks are prominent among natural systems, exhibiting structural characteristics often optimized for biological function. To reveal such features in highly-connected networks, we introduce a new network characterization determined by a decomposition of network-connectivity into low-rank and sparse components. Based on these components, we discover a new class of networks we define as *amalgamated* networks, which exhibit large functional groups and dense connectivity. Analyzing recent experimental findings on cerebral cortex, food-web, and gene regulatory networks, we establish the unique importance of amalgamated networks in fostering biologically advantageous properties, including rapid communication among nodes, structural stability under attacks, and separation of network activity into distinct functional modules. We further observe that our network characterization is scalable with network size and connectivity, thereby identifying robust features significant to diverse physical systems, which are typically undetectable by conventional characterizations of connectivity. We expect that studying the amalgamation properties of biological networks may offer new insights into understanding their structure-function relationships.

With advances in technology and mathematical theory increasingly facilitating the study of complex networks, recent experimental evidence suggests that many networks are more densely-connected than previously proposed. In the case of the macaque cerebral cortex, for example, highly-resolved tracer injections have revealed 36% more inter-areal connections than reported by past studies, yielding a total edge density of 66%^1^. Similarly, the application of new methodology in studying node interactions has led to the discovery of dense gene, protein, and food-web networks^2^,^3^,^4^,^5^. In general, network characterizations, such as small-worldness or scale-freeness, have proven useful in studying physical systems with sparse connectivity, such as in the framework of non-equilibrium physics, sociology, and biology^6^,^7^,^8^. In contrast to sparsely-connected networks, dense networks trivially have short path lengths and high clustering coefficients, making these common descriptors of connectivity unable to characterize their unique structure^8^,^9^.

For sparse networks, communities, composed of particularly densely-connected nodes, give significant insight into the functional roles of various inhomogeneous groups of nodes and the overall network structure-function relationship^10^,^11^,^12^,^13^. There has been a recent development of a host of community detection techniques, such as modularity maximization, edge betweenness, maximum likelihood, and graph-Laplacian spectral methods^14^,^15^,^16^,^17^, which are often successful in identifying communities in large networks. However, in the case of dense networks, the question of how to formulate the notion of communities remains a theoretical challenge important for understanding diverse biological systems^18^,^19^,^20^,^21^,^22^.

In this article, we introduce a new framework to characterize the distinct architectures of densely-connected networks through sparse and low-rank (SL) decomposition of connectivity (adjacency) matrices. The SL decomposition is a newly-developed method of recovering the low-dimensional linear structure in non-ideal observations by decomposing a data matrix into a low-rank component (signal) and a sparse component (noise or perturbation)^23^,^24^. It has amassed a wide range of applications, including feature recognition, video surveillance, and image denoising^25^,^26^,^27^. Using the SL decomposition, we discover a class of networks, i.e., *amalgamated* networks, that exhibits both modularity and dense connectivity. By analyzing a variety of real-world network data, we demonstrate that these networks naturally manifest in biological systems, and hypothesize their functional advantages under biological constraints^28^. We expect that the amalgamated network structure is evolutionarily desirable, with the modularity of amalgamated networks facilitating integrated network activity^29^ and the observed high connection-density promoting rapid communication among functional groups. Since these networks are also resilient to node attacks, preserving overall network structure and activity fundamental for biological function, they have likely remained pervasive in many physical systems and may give significant insight into their characteristics.

## Results

### Sparse and Low Rank Network Decomposition

In motivating the amalgamation characteristics, consider a densely-connected network of *n* nodes with connectivity matrix, *A*, containing an intermediate level of randomness. Such a network, due to its high degree, will comprise of a large number of cliques, which are sets of vertices in which every possible pair of nodes is connected, or near-cliques. For a network composed of disjoint cliques indexed such that the nodes in each clique are numbered in sequential order, each clique will contribute a block of 1’s along the diagonal of *A* and each block will increase the rank of *A* by 1. A network composed of *m* disjoint cliques will therefore have rank(*A*) = *m* < *n*, and thus *A* will be of low rank. Likewise, if a network is not complete yet dense, then there will typically also exist sparse edges connecting near-cliques. In this case, *A* can be decomposed into the sum of two components, namely low-rank matrix *L*, which contains the connections within near-cliques, and sparse matrix *S*, which contains the connections between near-cliques. As will be discussed below, the rank and density of these components are scalable with network size, statistically reliable, and can indeed be used as a diagnostic metric for describing topological features of such densely-connected networks.

To determine *L* and *S*, we devise a new method of decomposing unweighted connection matrices. First, we compute the connectivity matrix SL decomposition *A* *=* 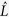 + *Ŝ* where 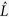 is low-rank and *Ŝ* is sparse. Intuitively, this is achievable by simultaneously minimizing the rank of 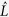 and sparsity of *Ŝ*. Because this problem is generally not solvable in polynomial time^23^, a more viable convex alternative is the optimization problem


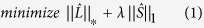


given 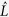 + *Ŝ* *=* *A*, where nuclear norm 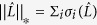 is the sum of the singular values, *σ*_*i*_ of 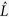. Intuitively, the number of nonzero singular values indicates the rank of 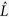. In addition, 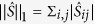 where *Ŝ* *=* (*Ŝ*_*ij*_). Note that the number of nonzero values in *Ŝ* indicates its sparsity. The sparsity penalization parameter λ can be regarded as the weight balancing the minimization of these two terms^23^. The parameter λ controls the number of zero entries in *Ŝ*, with higher λ yielding more sparse connectivity. In general, Problem (1) can be solved efficiently by algorithms such as singular value thresholding, augmented Lagrangian, and proximal gradient methods^27^,^30^,^31^, Sufficient conditions were proposed to address the existence and uniqueness of the solution to Problem (1), however, these conditions essentially require that the solution matrices, 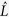 and *Ŝ*, demonstrate certain structure, such as incoherence and sparsity. In general, it is quite difficult to provide necessary conditions for the success of the SL decomposition based only on the structure of a given connectivity matrix^24^,^25^.

One difficulty of this approach is choosing the degree to which connections in *Ŝ* should be penalized, which can often only be done *a priori* for specific classes of components^25^. While in some cases λ can only be computed experimentally^23^,^32^,^33^, we instead devise a technique for choosing λ that is quite successful for our application. For all decompositions, we choose as our sparsity penalization parameter


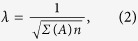


where Σ(*A*) is the density of *A*, defined as the fraction of nonzero elements of *A*, such that a completely connected network will have a density of 1. The scaling factor 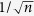 was established by Candés, reducing the penalization of sparse connections in larger networks, which have more possible connections^25^. In addition, we introduce a factor of 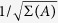 to conserve the penalization of connections in *Ŝ* regardless of the density of *A*. Without this new normalization, in the case of more sparsely-connected networks, for example, under-penalization of connections (i.e., with 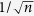 scaling only) in *Ŝ* may result in the sparse-component capturing all connections with none contained in 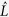. By using our choice of λ, a similar percentage of connections will be captured by *Ŝ* for networks with diverse connection densities.

**Figure 1:**
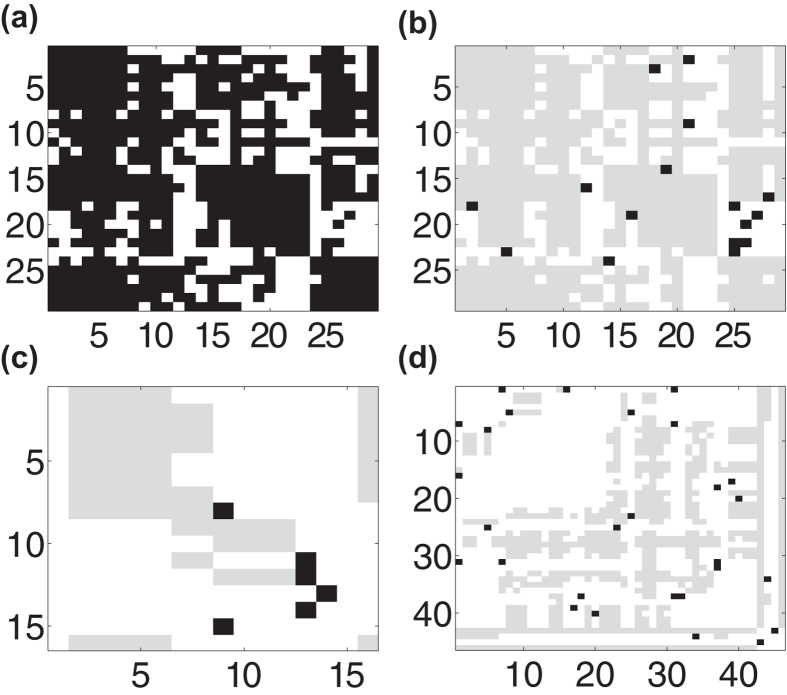
Amalgamated networks. (**a**) Connectivity matrix, *A*, for the macaque cerebral cortex network of *n* = 29 cortical areas^1^. The correspondence of nodes to cortical lobes is as follows: 1–7 (prefrontal), 8–13 (parietal), 14–19 (temporal), 20–23 (occipital), 24–28 (frontal), and 29 (limbic). Connections are marked in black. (**b**) The connectivity matrix is partitioned using the SL network decomposition into low-rank component *L* (grey) and sparse component *S* (black). (**c**,**d**) Gene regulatory and food-web network decomposition using the same color scheme as in (**b**). The relative Frobenius-norm error in the recovered network connectivity matrices are 0.0035, 0.0059, and 0.0167 for the cortical, gene, and food-web networks, respectively.

After completing the initial decomposition, we threshold each matrix element so all entries are 0 s or 1 s, reflecting the absence or presence of connections in an unweighted network, to yield decomposition components *L* and *S*. Our study indicates that the decomposition is insensitive for a range of thresholds. Note that because of this thresholding, the sum of the final decomposition, *A*_*r*_ = *L* + *S*, is an approximation of *A* which we show to be highly accurate in Fig. 1. It is also important to remark that the connections between nodes in each component are invariant with respect to node indexing, giving consistent network descriptors regardless of how nodes are labeled.

### Network Characterization

In characterizing a given network of *n* nodes, we analyze structure embedded by both the rank of *L* and sparsity of *S*. For the low-rank component, we compute the normalized rank of *L*, *ν*(*L*) = Rank(*L*)/*n*. In this way, full-rank matrices will have *ν*(*L*) = 1. Similarly, for the sparse component, we determine the density of *S*, Σ(*S*), the fraction of nonzero elements of *S*. Both metrics are bounded in [0,1], and are therefore useful in comparing networks of varying sizes.

**Figure 2:**
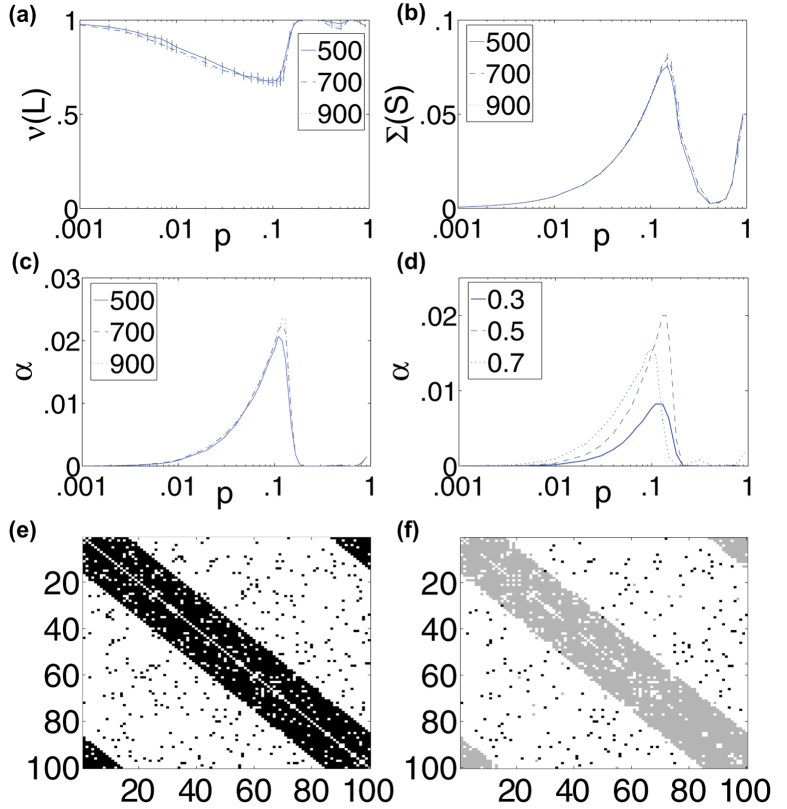
Amalgamation characteristics and connectivity matrix decomposition statistics. A family of networks is constructed using the WS method, varying rewiring probability, *p*. For each connectivity matrix, *A*, the network SL decomposition is computed. (**a**) *ν*(*L*) as a function of *p*. (**b**) Dependence of Σ(*S*) on *p*. (**c**) Amalgamation parameter, *α*, as a function of *p*. (**d**) Dependence of *α* on edge density. In (**a**)-(**c**), networks are of size *n* = 500, 700, and 900 nodes with an edge density of 66%. In (**d**) networks are of size *n* = 500 nodes with edge densities 30%, 50%, and 70%. For each plot, the mean value over an ensemble of 20 network realizations is depicted, with error bars corresponding to the standard deviation of the statistic. (**e**) Example connectivity matrix for a WS network with 100 nodes, edge density of 30%, and intermediate rewiring probability *p* = 0.1. (**f**) The connectivity matrix in (**e**) decomposed using the SL network decomposition into low-rank component *L* (grey) and sparse component *S* (black). The relative Frobenius-norm error in the recovered network connectivity matrix is 0.0042.

As an illuminating example, we examine the macaque cerebral cortex connectivity network depicted by Fig. 1 (a), taking the connections to be unweighted and the diagonal entries of *A* to be 1 under the assumption of intra-areal feedback^1^. Using the SL decomposition, we plot in Fig. 1 (b) the low-rank and sparse network components. We observe that the normalized rank of *L* is *ν*(*L*) = 0.69, which can be viewed as small compared to the minimum *ν*(*L*) of 0.68 for analogous WS networks shown in Fig. 2 (a), indicating the existence of modules in the anatomical cortical network. The presence of such modules suggests an organization of functional integration indicative of segregated neural processing in the brain^34^. The corresponding matrix *S* is very sparse, with Σ(*S*) = 0.019. Remarkably, 14 out of the total 16 connections in the sparse component are between different lobes in the cerebral cortex, which are each large clusters of cortical areas with common topological and functional features^1^. Thus, in accordance with our intuition, the *L* component typically captures the dense connections within functional modules of nodes, whereas the *S* component constitutes the connections between the modules.

In Fig. 2, we investigate the dependence of the network decomposition on the regularity of network topology, constructing a set of networks that interpolates between regular and random connectivity and analyzing the structure of both *ν*(*L*) and Σ(*S*) for each network. Using the Watts-Strogatz (WS) network construction, we first assemble a regular ring lattice network of *n* nodes with a high mean degree *k* such that each node is connected to *k*/2 neighbors on each side. Then, we rewire each edge with probability *p*, removing the original edge and randomly choosing with equal probability among all possible new edges to form a rewired connection. For networks with a high density of 66%, as in the cortical network, we plot the dependence of *ν*(*L*) and Σ(*S*) on *p* in Fig. 2 (a) and (b), respectively. For low values of *p*, which correspond to more regular networks, the large number of near-lattice connections in *A* produce a high-rank *L* and the very few rewires yield a highly sparse *S*. Hence, *ν*(*L*) is very high while Σ(*S*) is quite low. In contrast, for high *p*, the large number of unstructured connections in *A* corresponds to a high rank *L* with a high Σ(*S*) due to the large number of rewires. In contrast, using small-world measures, we instead observe that the average path length remains approximately constant and the clustering coefficient only decreases by 6% over the same set of rewiring probabilities, giving little indication of network topological changes.

According to our SL characterization, only for intermediate values of *p* do we observe unique topological features typically exhibited by densely-connected natural networks, such as the macaque cerebral cortex. In this region, *ν*(*L*) exhibits a clear local minimum, indicating the existence of many large modules, and simultaneously Σ(*S*) displays a local maximum, which is less than 8%, indicating sparse connections between modules. In Fig. 2 (c), we observe that this region corresponds to a global maximum of the amalgamation parameter, *α,* defined as


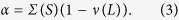


We refer to this class of dense networks as *amalgamated* networks, composed of large and highly-connected modules with relatively sparse interconnections. Connections within these modules are typically captured by the entries of *L*, whereas links exhibiting high-betweenness are primarily contained in *S*, which we have verified numerically. Therefore, *α* measures the degree of amalgamation exhibited by a network, quantifying the rank of *L* (modularity) and density of *S* (interconnections between modules). To demonstrate this intuition, we depict in Fig. 2 (e) the connectivity matrix for a WS network with intermediate rewiring probability *p* = 0.1 and an edge density of 30%, and display its corresponding SL decomposition in Fig. 2 (f). Clearly the low-rank component primarily captures the remaining ring-lattice connections after rewiring, and the sparse component mostly encapsulates the rewired connections linking the highly-connected clusters in *L*. We note that while a network composed of a small number of large disjoint cliques will have the lowest rank, such a network will still have a zero *α* value since Σ(*S*) = 0 in this case. Ideally, an amalgamated network is composed of a small number of interconnected near-cliques, resulting in a large 1 − *ν*(*L*) term from the low rank of the network and a relatively large Σ(*S*) from the connections between modules, which may be numerous but much less dense than the connections within modules.

In amalgamated networks, modules often act as integrated functional groups, which we will discuss in the context of several real-world networks in the following section. The information flow within such networks is facilitated via local spreading and global communication. Intuitively, *α* can be used to characterize the optimal information flow achievable by a proper balance between the number of modular structures and the number of the connections amongst them^35^,^36^,^37^.

To summarize, the general procedure for decomposing the network connectivity and subsequently characterizing the amalgamation properties is composed of four main steps. First, the network connectivity matrix is decomposed into low-rank and sparse components by solving optimization problem (1) with the penalization parameter λ chosen by Eq. (2). Then, the elements of the component matrices are thresholded to obtain an unweighted approximation of the original connection matrix. Third, the network amalgamation parameter defined in Eq. (3) is computed using the normalized rank of the low-rank component and density of the sparse component. Finally, the computed network amalgamation parameter may be compared to that of other networks or a baseline value to determine the extent to which the network may be considered amalgamated. We remark that, as shown for small *p* in Fig. 2, lattice networks have very small *α* values, as do networks with completely random connectivity. While we could define *α* such that it is normalized by the amalgamation for a benchmark network with random or regular connectivity, this would no longer guarantee that *α* remains bounded, and so in this work we directly compare *α* values as defined by Eq. (3) among networks to characterize amalgamation properties. Intuitively, one may view networks with *α* values comparable to WS networks with similar edge densities and intermediate rewiring probability, for which *α* is maximized as in Fig. 2, as amalgamated.

It is important to remark that amalgamated network properties scale well with network size and are stable across network realizations, allowing for direct comparison of topology regardless of variations in network size and small perturbations in connectivity. Fig. 2 (a–c) displays the average values of the network statistics across an ensemble of network realizations and the associated standard deviations. We observe that the particularly small error bars, especially for *α*, give a stable characterization of amalgamated networks. Likewise, since both factors in *α* are bounded in the interval [0 1], *α* is a strictly bounded characterization of amalgamation that will not increase with network size as in the case of other connectivity measures^38^. In Fig. 2 (a–c) we also plot the scaling of these statistics for networks of sizes, *n* = 500, 700, and 900 nodes, while holding the density of the connections constant at 66%. Regardless of network size, we observe a nearly identical amalgamation structure, demonstrating the robustness of the characterization.

For dense networks, with varying levels of connectedness, we find that the amalgamation properties scale relatively well and therefore facilitate fair comparison for networks of different densities. We plot in Fig. 2 (d) the average amalgamation parameter for networks with edge densities of 30%, 50%, and 70% over an ensemble of network realizations. We note that the maximum *α* occurs in approximately the same parameter regime for each density. Since we observe variation in *α* with respect to connection density, we remark that it is reasonable to compare amalgamation properties among networks with similar edge density. Furthermore, because amalgamated networks are so densely connected, their structure persists even with the removal of many nodes and edges. In an amalgamated network of *n* = 500 nodes with 66% edge density, for example, the random removal of 10% of the nodes and their corresponding edges only results in an approximately 2% reduction in *ν*(*L*) and 8% decrease in Σ(*S*), therefore having a relatively small impact on the network characterization.

### Real-World Networks

Analyzing real-world data, we find certain systems exhibit a varying degree of amalgamation properties. For example, the cortical network in Fig. 1 (a) is quite amalgamated with a high *α* = 0.006. In comparison, we note that for a 29 node WS network with the same connection density, the mean maximum *α* is 0.0017, and therefore the *α* value for the cortical network can be viewed as large. Corresponding to this amalgamation, we remark that the exhibited modularity and dense connectivity contribute to several important brain processes^29^. Considering interconnected networks undergo sharp transitions from independent to coupled nearly identical dynamics as more interconnections are formed^39^, the relatively sparse interconnections between functional modules control integration of compartmentalized cortical processes^34^, without inter-module connections so numerous that the dynamics of each module are nearly identical. Also, within the 6 relatively large functional regions considered, dense intra-areal connections promote rapid parallel processing necessary for sensory integration over time-scales small enough for survival^40^,^41^.

Similarly, in the Escherichia coli glycolysis gene network of *n* = 16 nodes with edge density 33% in Fig. 1 (c) 2, *ν*(*L*) = 0.25, Σ(*S*) = 0.023 and *α* = 0.018. Composed of a small number of relatively large clusters of nodes, we observe that the gene network exhibits even more amalgamation than the cortical network, with a markedly higher *α* value. The gene network also displays a modular structure with an intuitive biological interpretation. Considering these genes have both fast and slow interactions, we emphasize that 5 of the total 6 sparse connections include fast gene-interactions while the low-rank component, on the other hand, mostly includes densely-connected slow gene-interactions. These interactions facilitate various feed-back loops necessary for the interplay between slow protein synthesis and fast enzymatic reactions crucial for carbon assimilation^2^. Likewise, numerous experimental studies have provided strong evidence of modularity in gene interactions akin to amalgamated networks^42^.

Although some food-webs exhibit small-world structures, most do not because their edge density is relatively high despite containing few large clusters of nodes^43^. Therefore, we investigate if amalgamation characteristics can instead be used to describe such networks. In the case of the Mondego estuary food-web network with *n* = 46 nodes and an edge density of 34% in Fig. 1 (d)^4^, we observe amalgamation, but to a lesser extent than the other example networks, with *ν*(*L*) = 0.76, Σ(*S*) = 0.013, and *α* = 0.0032. This is likely because while the food-web network still is composed of several near-cliques, the average cluster of nodes is relatively small in size. Observing a modular structure similar to other biological networks, the sparse connections in this case often connect organisms of differing trophic levels belonging to distinct ecological communities. Such modular structure is widely observed among food-webs, and is hypothesized to sustain ecosystems against environmental disturbances, such as natural disasters and extinctions^44^,^45^. Comparing these network characterizations, we see that the amalgamation properties are able to describe topological variations and provide a useful diagnostic for detecting differences in the structure of densely-connected networks, which would typically be missed by classical descriptors of network connectivity.

## Discussion

In summary, we have developed a framework for characterizing the connectivity structure of densely-connected networks using a decomposition of network connectivity matrices into sparse and low-rank components. Based on the density and rank of these respective components, we then measure the network amalgamation, *α*, a novel descriptor of both the modularity and interconnectedness of a network. In addition, we identify a class of networks with particularly high *α* as amalgamated networks, which we show to be prominent in diverse biological systems. We hypothesize that such networks arise naturally due to their advantageous properties, including fast node communication, functional modularity, and structural stability.

We remark that our characterization methodology is well-suited to be extended to formulate alternative network characterizations. In the case of weighted networks, our method could be adapted by rounding the entries of each component to the nearest appropriate weight. Likewise, due to the stability and scalability of *α*, we could instead define an amalgamated network by thresholding on a specific high value of *α* given a certain level of connection density. For generality, to further characterize amalgamation properties, we instead compare the *α* values of real-world networks and highly-amalgamated WS networks of similar density. In this framework, real-world networks with higher *α* than corresponding WS networks may be considered amalgamated. We note that through direct comparison, *α* values may also be used to distinguish among the randomness of connectivity for densely-connected networks similar to how small-world measures are applied in the case of sparsely-connected networks^8^,^38^.

It is important to emphasize that community structure itself is no guarantee of amalgamation. In addition to modularity, dense overall connectivity, useful for rapid functional integration, and yet sufficiently sparse interconnectivity between modules, facilitating functional segregation, are necessary for amalgamation. A network may contain several communities, but still lack an approximate low-rank structure due to excessively dense inter-community connections, thereby preventing it from being characterized as amalgamated. Likewise, a network instead composed of large cliques may have insufficient connections between these cliques for amalgamation. Considering that the current tools of network science make it difficult to identify communities in densely-connected networks^18^,^19^,^20^,^21^,^22^, the framework we introduce is particularly useful in gaining novel insight into dense network structure and may also further characterize networks for which it is possible to separate modules from sparse inter-module connections using community detection. Likewise, it may also be informative to use amalgamation properties to similarly characterize sparsely-connected networks and determine how these properties compare to conventional measures of connectivity. In light of the prevalence of dense connectivity in biological networks, we hope that our work will be useful in understanding those networks and the importance of connection density to network function.

## Additional Information

**How to cite this article**: Barranca, V. J. *et al*. A Novel Characterization of Amalgamated Networks in Natural Systems. *Sci. Rep.* **5**, 10611; doi: 10.1038/srep10611 (2015).
